# A Genome for Edith’s Checkerspot Butterfly: An Insect with Complex Host-Adaptive Suites and Rapid Evolutionary Responses to Environmental Changes

**DOI:** 10.1093/gbe/evac113

**Published:** 2022-07-25

**Authors:** Kalle Tunstrom, Christopher W Wheat, Camille Parmesan, Michael C Singer, Alexander S Mikheyev

**Affiliations:** Department of Zoology, Stockholm University, Stockholm, Sweden; Department of Zoology, Stockholm University, Stockholm, Sweden; Station d’Écologie Théorique et Expérimentale, CNRS, 2 route du CNRS, 09200 Moulis, France; Biological and Marine Sciences, University of Plymouth, Plymouth, UK; Department of Geological Sciences, University of Texas at Austin, TX, USA; Station d’Écologie Théorique et Expérimentale, CNRS, 2 route du CNRS, 09200 Moulis, France; Biological and Marine Sciences, University of Plymouth, Plymouth, UK; Research School of Biology, Australian National University, Canberra, ACT, Australia

**Keywords:** genome, long-read sequencing, HiC scaffolding, climate-change model

## Abstract

Insects have been key players in the assessments of biodiversity impacts of anthropogenically driven environmental change, including the evolutionary and ecological impacts of climate change. Populations of Edith’s Checkerspot Butterfly (*Euphydryas editha*) adapt rapidly to diverse environmental conditions, with numerous high-impact studies documenting these dynamics over several decades. However, studies of the underlying genetic bases of these responses have been hampered by missing genomic resources, limiting the ability to connect genomic responses to environmental change. Using a combination of Oxford Nanopore long reads, haplotype merging, HiC scaffolding followed by Illumina polishing, we generated a highly contiguous and complete assembly (contigs *n* = 142, N50 = 21.2 Mb, total length = 607.8 Mb; BUSCOs *n* = 5,286, single copy complete = 97.8%, duplicated = 0.9%, fragmented = 0.3%, missing = 1.0%). A total of 98% of the assembled genome was placed into 31 chromosomes, which displayed large-scale synteny with other well-characterized lepidopteran genomes. The *E. editha* genome, annotation, and functional descriptions now fill a missing gap for one of the leading field-based ecological model systems in North America.

**Significance**

Edith’s checkerspot, *Euphydryas editha*, is a nonmigratory butterfly that exhibits a remarkably high degree of phenotypic variation among populations and ecotypes. For this reason, it has become a model system for understanding how species and populations can rapidly adapt to changes in their local environment. However, the lack of genomic resources has made investigating both the genomic basis of these traits as well as the genetic consequences of this rapid adaptation impossible. Here we present a high-quality, chromosome level reference that will aid researchers pursuing these questions.

## Introduction

Despite the prominence of insects in studies of human impacts on nature, there is surprising disagreement over the extent and importance of anthropogenic influences (Gonzalez et al. 2016; Macgregor et al. 2019). Two recent papers exemplify this debate in the community. On the one hand, Sánchez-Bayo and Wyckhuys (2019) report “insectageddon,” catastrophic global-scale declines in insect biomass, abundance, and diversity that predict extinction of 40% of species in the coming decades. On the other hand, Deutsch et al. (2018) predict that insects will profit from climate warming. In general, scientific community seems to be struggling, both to determine what human activities have already done to insects and to predict what their future impacts will be (Parmesan et al. 2022). One fundamental question is: how should impacts be assessed? The level at which biodiversity loss is measured (e.g., species, subspecies, ecotypes, or genotypes), and the metric by which loss is measured (e.g., changes in abundance, total area occupied, or population extinctions of particular ecotypes and associated genotypes) both impact our ability to identify and respond to biodiversity loss. Given this complexity, one way forward to assess these impacts is to focus upon species representing different patterns of geographical variation and local adaptation. Additionally, an ideal species would also exhibit extensive ecotypic variation that shows a mix of endangered and unthreatened populations. Within this category, it would be useful that the target species is well-studied, with known variation in population dynamics, local adaptation, and ecological interactions. To this end, we present a chromosome level genome assembly for Edith’s Checkerspot Butterfly, *Euphydryas editha*.

*Euphydryas editha* is a nonpestiferous, nonmigratory species, distributed across the western USA and from Baja California to central Alberta. It has evolved a geographic mosaic of ecotypes differing in adult size, phenology, habitat choice, and host preference (McBride and Singer 2010; Singer and McBride 2010, 2012), with these ecotypes exhibiting such strong local adaptation that populations can differ significantly in these heritable traits over distances as short as 20 km. Some of these ecotypes have stable populations, whereas others show a dynamic of extinctions and recolonization (Ehrlich et al. 1980). Populations can be small and isolated, occupying one or two hectares, or they can exist as components of meta-populations extending over 20–100 km^2^ (Harrison et al. 1988; Thomas et al. 1996: 19). Individual populations of *E. editha* have repeatedly demonstrated their ability to evolve rapidly in response to local environmental change (Singer and Parmesan 2018, 2019). Several ecotypes are sensitive to climate change (Parmesan and Singer 2022), as a result of which *E. editha* at the species level was already showing the expected latitudinal and altitudinal range shifts in the early 1990s (Parmesan 1996). Many subspecies of *E. editha* have been named, for which the principal (usually the only) criterion has been wing-pattern phenotype of adults. Some subspecies are congruent with ecotypic variation, and some are not. Three of these subspecies, the Bay Checkerspot, the Quino Checkerspot, and Taylor’s Checkerspot, are federally endangered and currently subjects of conservation efforts.

Despite the decades of ecological and evolutionary field studies briefly reviewed above, there has been relatively little genetic work done on this species, and it has been limited to mitochondrial, microsatellite, and Amplified Fragment Length Polymorphism (AFLP) studies (Mikheyev et al. 2013; Parmesan et al. 2015; Singer and Parmesan 2021). The nonexistence of genomic resources has meant that *E. editha*, whereas being a promising model for the study of the genomic architecture of adaptation and decline, has not been used as such. Here we remedy this technical gap by providing a high-quality genome for future investigations.

## Results and Discussion

### Assembly

**Fig. 1.**
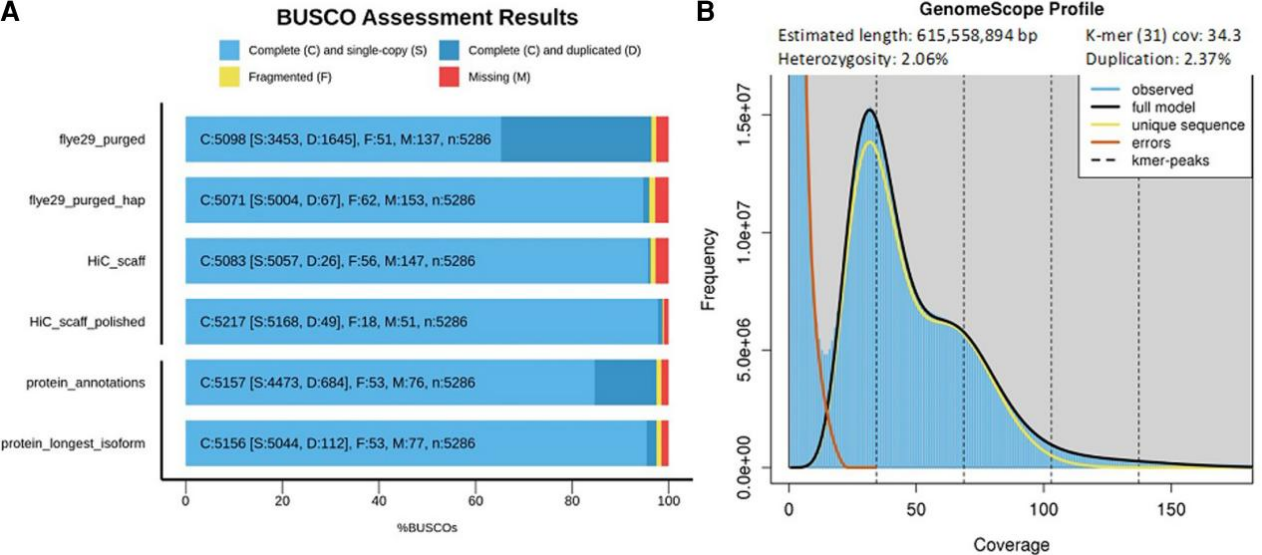
Genome assembly assessment for the *E. editha* butterfly, showing improvements during genome refinement steps, the annotation, and an estimate of genome size. (*A*) Assessment of the content and quality of 5,286 single copy orthologs within Lepidoptera, beginning with the initial genome assembly (fly29_purged), the result of merging the genome down to a haploid copy (fly29_purged_hap; note the decrease in the number of duplicated genes D), the HiC scaffolded genome, and the final polished version (HiC_scaff_polished). After these are the BUSCO results upon the protein sets generated from the genome annotation, for all proteins including isoforms (protein_annotation), as using only the longest isoform per locus in the annotation (protein_longest_isoform). (*B*) Genome size estimate using k-mer counting of Illumina sequence data, showing the estimated genome size, heterozygosity, k-mer coverage, and duplication rate.

**Fig. 2.**
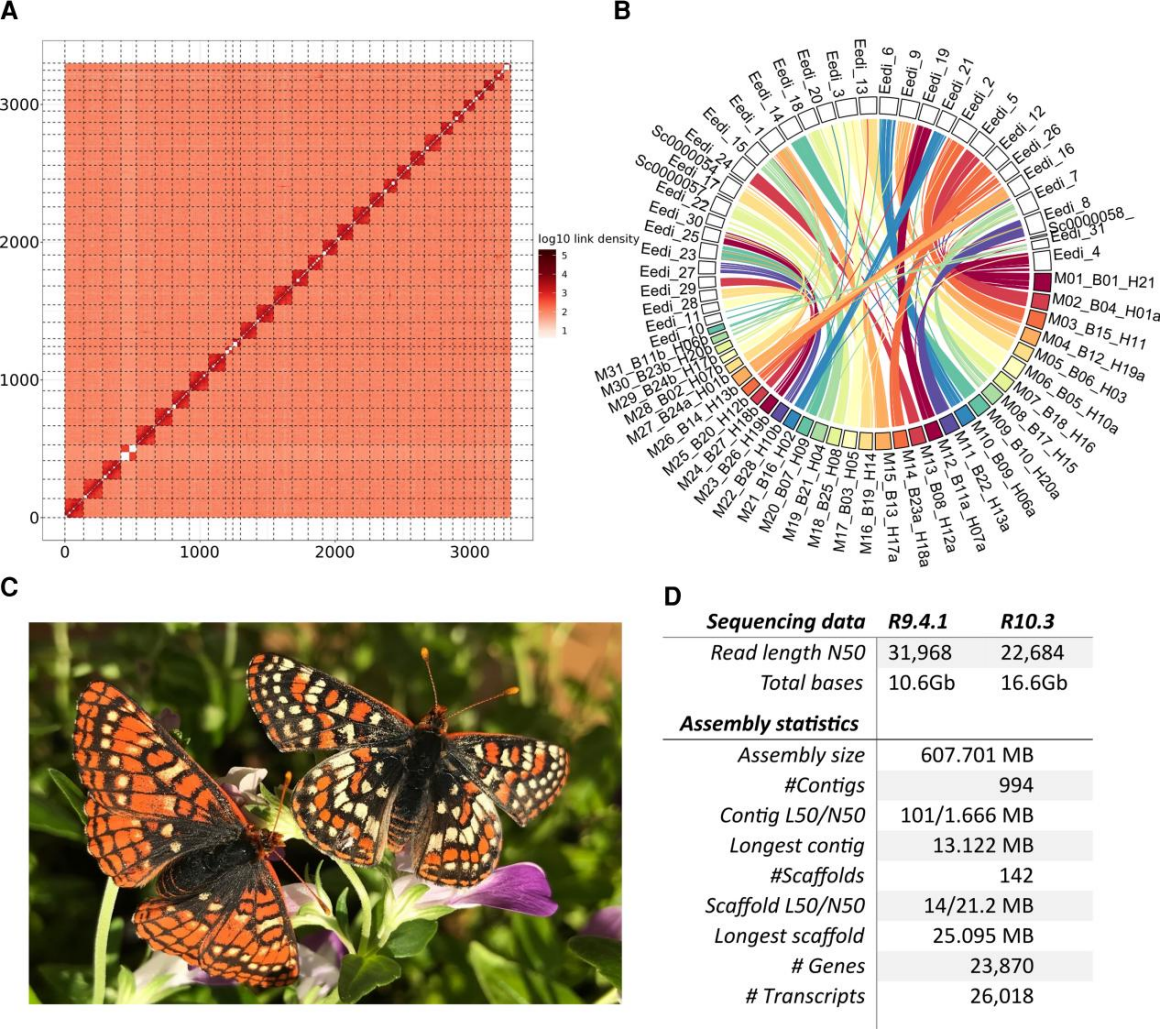
Assessment of genome contiguity, showing Hi-C scaffolding results and whole genome alignment to related species. (*A*) Hi-C interaction matrix of the ordered scaffolds along the 31 chromosomes (*B*) Circos plot of whole genome alignment between *M. cinxia* chromosomes (colored blocks along outer edge) to *E. editha* chromosomes (noncolored blocks), with regions of inferred orthology indicated as colored lines between them. For example, M01_B01_H21 in maroon is *M. cinxia* chromosome 1, which corresponds to *B. mori* chromosome 1, and *H. melpomene* chromosome 21. These are all Z chromosomes in these species. This corresponds to *E. editha* scaffold 4 (Eedi_4). Each of the maroon lines connecting these two is a genomic region of alignment. This harmonic plot, of all colored lines primarily extending between single chromosomes of both species is consistent with the highly conserved nature of chromosome evolution in the Lepidoptera. The small discrepancies are likely repetitive content (or low frequency translocation events). (*C*) Example of phenotypic variation between a female *E. editha* from Rabbit meadow (left) and a male *E. editha* from Tamarack (right). (*D*) *Table of* sequencing and assembly summary statistics.

Using 27.2 Gb of ONT long-read data (after filtering Qscore of 9, R9.4.1 flowcell N50 read length = 31,968 of 10.6 Gb data, R10.3 N50 of 22,648 of 16.6 Gb), we assembled a moderately contiguous *E. editha* draft assembly using Flye, with purged haplotypes (N50 = 1,388,817 bp, contigs = 1,747, total = 801 Mb), which had a high content of complete BUSCOs, albeit with a very high duplication rate (fig. 1*A*). After haplomerging this genome, we significantly increased N50, whereas reducing the number of contigs, genome size, and BUSCO duplication levels (N50 = 1,752,737 bp, contigs = 994, total = 608 Mb; fig. 1*A*), indicating a much improved, haploid version of the genome that was much closer in size to our k-mer based estimate (fig. 1*B*). The genome was then scaffolded to chromosome scale using Hi-C data, which corrected a few assembly errors and placed 98% of the assembly (597,781,036/607,788,004) onto 31 chromosomes (fig. 2*A*), which is close to other species in the genus, which vary from 30 to 31 (Robinson 1971). This chromosome was then polished using 133× coverage of 10× Illumina sequencing data, resulting in a final high-quality genome assembly (N50 = 21,225,494 bp, contigs = 142, total = 607.8 Mb; fig. 1*A*). Red detected and masked 41% repetitive content.

### Annotation

Our annotation identified 23,870 genes producing 26,018 transcripts (25,611 of which started and ended with start- and stop-codon and had no internal stop-codons). The annotation contained 97.5% of expected BUSCO genes, which had a high number of duplicates due to isoforms (fig. 1*A*). Filtering of the annotation to removed overlapping genes and retain only the longest isoforms of each gene, reduced the number of duplications from 12.9% to 2.1% (fig. 1*A*). Functional annotation of the assembly was performed using EggNOG-mapper (v2.1.7) comparing it against the EggNOG database v5 and integrated into the annotation GFF.

### Synteny Assessment

To assess the accuracy of our genome assembly and chromosomal assignment, we conducted a whole genome alignment to the closest relative with assembly at the chromosome scale, which was *Melitaea cinxia* (Nymphalidae, Lepidoptera) (fig. 2*B*). Between the two species, which last shared a common ancestor ∼27 Ma, there do not appear to be any large-scale structural rearrangements (Chazot et al. 2021). Importantly, in our alignment, the naming of the *M. cinxia* chromosomes also indicates their orthologs in another nymphalid butterfly, *Heliconius melpomene*, as well as the moth, *Bombyx mori*, further highlighting the standard nature of the chromosomal organization in *E. editha* (fig. 2*B*).

## Conclusion

Here we present our assembly for *E. editha*, an established model system for studying geographic mosaics of ecological adaptation and rapid evolutionary responses to anthropogenically driven environmental change. Our assembly placed 98% of the 608 Mb genome into a chromosomal framework and exhibited exceptionally high and accurate gene content as measured using BUSCO, placing this species among the best assembled Lepidopteran genomes to date (Ellis et al. 2021). We were able to annotate 23,870 high-quality genes and provide functional information for 20,771 of these. In comparison to another chromosome-scale butterfly genome, we verify that our assembly is not only highly contiguous but accurately assembled as there is high synteny across all of the 31 chromosomes shared between these species (Hill et al. 2019; Smolander et al. 2022). This work provides the foundation upon which detailed study of the eco-evolutionary dynamics of this focal species and its endangered subspecies can now develop. Further, given the extensive literature documenting multi-trait hostplant adaptations in this species, identification of the genomic regions involved can now progress using a wide range of population genomic tools. In sum, this genome will serve as a valuable resource to a diverse community of researchers.

## Materials and Methods

### Genome Sequencing

*Euphydryas editha* individuals were collected from Rabbit Meadow, CA (lat. 36.710, long. −118.373, elev. 2380 m). The female individual used for assembly was stored in 95% ethanol and kept frozen until extraction. High-molecular weight genomic DNA was extracted from the front half of the thorax with most of the cuticle removed using standard protocol for paramagnetic nanodiscs (Nanobind Tissue Big DNA kit, Circulomics). Before extraction, the ethanol was removed, and the tissue was rehydrated by soaking it in ethanol removal buffer (400 mM NaCl, 20 mM Tris, pH 7.5, and 30 mM EDTA). The isolated DNA was split into two aliquots and prepared for sequencing separately. Each aliquot was individually treated with Short Read Eliminator (SRE) or SRE XL (both from Circulomics), to selectively precipitate high-molecular weight DNA (>10 and >20 kb fragments, respectively). Isolated and size selected DNA were sequenced on MinION platform using two flowcells (R9.4.1 for the SRE XL size selected sample, and one R10.3 for the SRE treated sample) using ligation-based library prep LSK110. Once sequencing was finished, the raw reads were base-called using Super High Accuracy base-calling mode in GUPPY (v.5.0.2) software.

### Assembly

From the base-called reads, we assembled a draft genome assembly using Flye v2.9 using the default settings for nanopore reads base-called with super high accuracy mode (nano-hq) followed by two iterations of polishing with Flye (Kolmogorov et al. 2019). Haplotype redundancies were identified and purged from the draft assembly using Purge_dups v1.2.5, default settings (Guan et al. 2020), followed by Haplomerger2 v.20180603 (Huang et al. 2017). Contiguity and completeness of the assembly were evaluated after each step using stats utility in bbtools and BUSCO v.4.1.2 and the lepidoptera_odb10 database (Seppey et al. 2019). Genome size was estimated using GenomeScope (Vurture et al. 2017), with Jellyfish v. 2.2.10 (Marçais and Kingsford 2011) for k-mer counting (k-mer cutoff of 10,000), using Illumina paired end sequenced data (150 bp read length), prepared using chromium-linked reads technology from a separate individual. Note that linking adapters were trimmed using longranger basic before use v.2.2.2 (Marks et al. 2019).

### HiC Scaffolding

Chromatin conformation capture data were generated using a Phase Genomics (Seattle, WA, USA) Proximo Hi-C 2.0 Kit, which is a commercially available version of the Hi-C protocol (Lieberman-Aiden et al. 2009). Following the manufacturer’s instructions, intact cells from two samples were crosslinked using a formaldehyde solution, digested using the DPNII restriction enzyme, end repaired with biotinylated nucleotides, and proximity ligated to create chimeric molecules composed of fragments from different regions of the genome that were physically proximal in vivo, but not necessarily genomically proximal. Continuing with the manufacturer’s protocol, molecules were pulled down with streptavidin beads and processed into an Illumina-compatible sequencing library. Sequencing was performed on an Illumina HiSeq, generating a total of 465 M PE150 read pairs. Reads were aligned to the draft assembly (fly29_purged_hap), following the manufacturer’s recommendations, using BWA–MEM (Li 2013) with the -5SP and -t 8 options specified, and all other options default. SAMBLASTER (Faust and Hall 2014) was used to flag PCR duplicates, which were later excluded from analysis. Alignments were then filtered with Samtools (Li et al. 2009), using the -F 2304 filtering flag to remove nonprimary and secondary alignments. Putative misjoined contigs were broken using Juicebox (Durand et al. 2016) based on the Hi-C alignments. A total of 13 breaks in 12 contigs were introduced, which was then followed by repeating the same alignment procedure on the resulting corrected assembly. Phase Genomics’ Proximo HiC genome scaffolding platform was then used to create chromosome-scale scaffolds from the corrected assembly as described in Bickhart et al. (2017). As in the LACHESIS method (Burton et al. 2013), this process computes a contact frequency matrix from the aligned Hi-C read pairs, normalized by the number of DPNII restriction sites (GATC) on each contig, and constructs scaffolds in such a way as to optimize expected contact frequency and other statistical patterns in HiC data. Approximately 60,000 separate Proximo runs were performed to optimize the number of scaffolds and scaffold construction in order to make the scaffolds as concordant with the observed HiC data as possible.

### Short Read Polishing

DNA from a second *E. editha* female captured at the same location (2018) was extracted using KingFisher Cell and Tissue DNA Kit from ThermoFisher scientific (N11997) using the robotic Kingfisher Duo Prime purification system. DNA quality was assessed using 260/280 ratio (Nanodrop 8000 spectrophotometer; Thermo Scientific, MA, USA) and the concentration was quantified on a Qubit 2.0 Fluorometer (dsDNA BR; Invitrogen, Carlsbad, CA, USA). 10×-chromium-linked read library preparation and sequencing were performed by SciLifeLab (Stockholm, Sweden). Strand specific barcodes were trimmed using Longranger basic and the reads aligned to the reference genome using BWA–MEM and polished using Polca from MaSuRCA v.4.0.8 (Zimin and Salzberg 2020). Repetitive content was identified and softmasked from the genome using RED v.05/22/2015 (Girgis 2015).

### Annotation

We used the Braker2 automated annotation pipeline to generate a comprehensive annotation of protein coding genes in the final assembly. We ran Braker2 in the genome and protein mode, using reference proteins from the Arthropoda section of OrthoDB v.10 (Lomsadze et al. 2005; Stanke et al. 2006, 2008; Gotoh 2008; Iwata and Gotoh 2012; Buchfink et al. 2015; Hoff et al. 2016, 2019; Brůna et al. 2020, 2021). Filtering of genome annotation to the longest isoform used scripts from the AGAT suite of tools v.0.5.1 (Dainat et al. 2022), including agat_convert_sp_gxf2gxf.pl, agat_sp_keep_longest_isoform.pl, and agat_sp_extract_sequences.pl. The resulting annotation was assessed based upon number of complete genes and BUSCO scores, for both all proteins and longest isoforms per locus. We assigned gene names and function to our predicted genes using eggNOG-mapper v.2 (Huerta-Cepas et al. 2019; Cantalapiedra et al. 2021).

### Synteny

We used nucmer (MUMmer4, v.4.0.0beta2) (Marçais et al. 2018) to align our final assembly to the chromosome level assembly of the closely related ecological model species, *M. cinxia* (Smolander et al. 2022), with scaffold naming incorporating chromosomal orthology with *B. mori* and *H. melpomene*. Synteny was visualized in R with the packages circlize v 0.4.12 (Gu et al. 2014) and RColorBrewer v1.1-2, (Neuwirth and Neuwirth 2014), using a set of custom bash and R scripts.

## Data Availability

The final genome assemblies and annotations have been archived on ENA, under the project number PRJEB51552, as an EMBL flat file containing both the genome fasta and annotation information (which can be extracted using a script in the AGAT software suite, agat_convert_embl2gff.pl, Dainat et al. 2022). Also available on ENA are the ONT MinION fastq sequences used for assembly (accession number ERR9284036, ERR9284037), the Illumina 10× data used for genome size estimation and polishing (accession number ERR9251014), as well as the Hi-C data used for scaffolding (ERR9285110). The Bash and R scripts for circos plotting follow previous work (Steward et al. 2021) and provided as supplemental information (SI) at https://github.com/rstewa03/Pieris_macdunnoughii_genome.
